# Genetic Diversity and Potential Virulence of *Listeria monocytogenes* Isolates Originating from Polish Artisanal Cheeses

**DOI:** 10.3390/foods11182805

**Published:** 2022-09-11

**Authors:** Renata Pyz-Łukasik, Waldemar Paszkiewicz, Michał Kiełbus, Monika Ziomek, Michał Gondek, Piotr Domaradzki, Katarzyna Michalak, Dorota Pietras-Ożga

**Affiliations:** 1 Department of Food Hygiene of Animal Origin, Faculty of Veterinary Medicine, University of Life Sciences in Lublin, Akademicka 12, 20-033 Lublin, Poland; 2 Department of Experimental Hematooncology, Medical University of Lublin, Chodźki 1, 20-093 Lublin, Poland; 3 Department of Quality Assessment and Processing of Animal Products, University of Life Sciences in Lublin, Akademicka 13, 20-950 Lublin, Poland; 4 Department of Epizootiology and Clinic of Infectious Diseases, Faculty of Veterinary Medicine, University of Life Sciences in Lublin, Głęboka 30, 20-612 Lublin, Poland

**Keywords:** *Listeria monocytogenes*, virulence genes, whole-genome sequencing, artisanal cheeses

## Abstract

Artisanal cheeses can be sources of *Listeria monocytogenes* and cause disease in humans. This bacterial pathogen is a species of diverse genotypic and phenotypic characteristics. The aim of the study was to characterize 32 isolates of *L. monocytogenes* isolated in 2014–2018 from artisanal cheeses. The isolates were characterized using whole genome sequencing and bioinformatics analysis. The artisanal cheese isolates resolved to four molecular groups: 46.9% of them to IIa (1/2a-3a), 31.2% to IVb (4ab-4b-4d-4e), 12.5% to IIc (1/2c-3c), and 9.4% to IIb (1/2b-3b-7). Two evolutionary lineages emerged: lineage II having 59.4% of the isolates and lineage I having 40.6%. The sequence types (ST) totaled 18: ST6 (15.6% of the isolates), ST2, ST20, ST26, and ST199 (each 9.4%), ST7 and ST9 (each 6.3%), and ST1, ST3, ST8, ST16, ST87, ST91, ST121, ST122, ST195, ST217, and ST580 (each 3.1%). There were 15 detected clonal complexes (CC): CC6 (15.6% of isolates), CC9 (12.5%), CC2, CC20, CC26, and CC199 (each 9.4%), CC7 and CC8 (each 6.3%), and CC1, CC3, CC14, CC87, CC121, CC195, and CC217 (each 3.1%). The isolates were varied in their virulence genes and the differences concerned: *inl*, *actA*, LIPI-3, *ami*, *gtcA*, *aut*, *vip*, and *lntA*.

## 1. Introduction

*Listeria monocytogenes* is a foodborne pathogen that is a serious and constant threat to public health worldwide, as documented in annual official reports from many countries and in other publications [1,2]. This pathogen has been associated with both sporadic episodes and large outbreaks of human listeriosis. Although the incidence rate is low (0.42 per 100,000 population), the disease commonly results in severe clinical outcomes, with high hospitalization (97.1%) and mortality rates (13%) [1]. According to the last European Food Safety Authority (EFSA) and European Centre for Disease Prevention and Control (ECDC) report, the highest notification rates were observed in Finland, Slovenia, Malta, and Sweden (1.7, 1.2, 0.97, and 0.85 cases per 100,000 population, respectively) and the lowest notification rates were reported by Romania, Bulgaria, Croatia, Ireland, Slovakia, Czechia, Poland, and Greece (≤0.19 per 100,000 population). In 2020, data from the 27 European Union (EU) member states gave the number of confirmed cases of invasive listeriosis in humans as 1876, including 62 cases in Poland. In that year, the numbers of hospitalizations and deaths were 780 and 167, respectively [1]. Listeriosis may occur in a non-invasive form and an invasive form. The non-invasive form of the disease is found in adults with competent immune systems and takes a mild course with flu-like symptoms and/or inflammation of the stomach and intestines. In contrast, the invasive form of the disease affects the young, old, pregnant, and immunocompromised segments of the population (the YOPIS risk group) and takes a grave, life-threatening course. The most common clinical forms of listeriosis include sepsis, meningitis, encephalitis (or other infections of the central nervous system), and in pregnant women, miscarriage, premature birth, stillbirth, or death of the fetus [3,4].

This pathogen is characterized by great genetic diversity and variability in virulence potential [5]. Consequently, *L. monocytogenes* isolates originating from food belong to different molecular groups, evolutionary lineages, sequence types (ST), and clonal complexes (CC) [6,7,8,9]. Due to differences in the virulence potential of *L. monocytogenes*, not all strains are equally capable of causing human infection. Many virulence factors participate in the pathogenicity of *L. monocytogenes* and allow it to infect, survive in, and replicate in a variety of host cell types [5]. Subgroups of this pathogen have different virulence phenotypes that may be associated with niche specificity [9]. Assessing the genetic diversity of *L. monocytogenes* is critical to understanding the epidemiology, ecology, and pathogenicity of this bacterium [10].

Artisanal cheeses have been produced continuously in many countries of the world. This type of foodstuff is produced in some regions of those countries by traditional methods (no advanced technological solutions are used nor production standards established for the process) and according to long-standing recipes indigenous to each region. Artisanal cheeses are appreciated by consumers around the world [11] and have cultural, social, and economic importance [12]. According to the literature, artisanal cheeses were vehicles of *L. monocytogenes* [13,14,15,16] and the cause of listeriosis outbreaks [17,18,19]. Reporting foodborne outbreaks is mandatory under Zoonoses Directive 2003/99/EC. The data of EFSA represent the most comprehensive set of data available at the EU level for assessing their public health burden, including those caused by *L. monocytogenes* [1]. In 2020, 16 listeriosis outbreaks were noted, and in one case, cheese was the vehicle for the outbreak [1]. The same report revealed that the prevalence rates of *L. monocytogenes* in various types of cheeses were in the range of 0.29–1.4% [1]. In contrast, artisanal cheeses from some countries were markedly more frequently contaminated, and *L. monocytogenes* was present in 4.1–30% of samples [13,15,16,20].

Addressing the pathogen’s complexity and variability, the aim of the study was to characterize *L. monocytogenes* isolates originating from Polish artisanal cheeses in terms of the molecular groups (including serogroups), evolutionary lineages, sequence types, clonal complexes, and the presence of virulence genes from among a set of 40.

## 2. Materials and Methods

### 2.1. Sampling

Artisanal cheeses contaminated with the pathogen came from 16 cheese dairies located in four administrative divisions in southern Poland (Table 1). *Listeria monocytogenes* were isolated from artisanal cheeses (hard cheeses *n* = 29; cottage cheeses *n* = 3) in the years 2014–2018. A total of 32 isolates were investigated. The presence of the pathogen was determined according to the methodology of the International Organization for Standardization (ISO) standards [21,22].

### 2.2. Whole-Genome Sequencing

#### 2.2.1. DNA Extraction

A single colony of bacteria was suspended in 100 µL of Tris-HCl (1 M, pH 8.0, RNase/DNase-free; EURX). To cell suspension, 20 µL of Lysozyme (concentration 10 mg/mL; A&A Biotechnology) and 10 µL of Lysostaphin from Staphylococcus staphylolyticus (concentration 2 mg/mL; Sigma-Aldrich, St. Louis, MO, USA) was added, then incubated for 30 min at 37 °C. DNA extraction was performed using an automated Maxwell RSC system (Promega) and Cell Culture Maxwell kit (Promega) according to the manufacturer’s instructions. Elution of DNA was performed in 100 µL of Tris-HCl (1 M, pH 8.0, RNase/DNase-free; EURX). The quantity and quality of DNA were evaluated by fluorometric (Qubit 3.0, Thermo Fisher Scientific, Waltham, MA, USA) and spectrophotometric (NanodropOne, Thermo Fisher Scientific) methods. Additionally, the integrity of isolated DNA was confirmed by capillary electrophoresis (DNF–488 High Sensitivity Genomic DNA Analysis Kit, Fragment Analyzer, Agilent, Santa Clara, CA, USA).

#### 2.2.2. Library Preparation

Libraries were prepared with the use of an automated pipetting station (Biomek i5; Beckman Culter, Indianapolis, IN, USA). For library construction, 100 ng of genomic DNA was enzymatically fragmented with the use of a KAPA Frag Kit (Roche) for 15 min at 37 °C. Then, the procedure was continued with a KAPA HypPlus kit (Roche, Basel, Switzerland) and KAPA DI Adapter Kit (Roche) according to the manufacturer’s recommendation. The quality and quantity of libraries were confirmed by capillary gel electrophoresis (DNF-473 Standard Sensitivity NGS Fragment Analysis Kit, Fragment Analyzer, Agilent, Santa Clara, CA, USA) and fluorimeter (BR/HS assay kit, Qubit 3.0, Thermo Fisher Scientific). 

#### 2.2.3. Sequencing

The Paired-end (2 × 300 bp) sequencing was performed using the V3 kit (Illumina, San Diego, CA, USA) and a Miseq platform (Illumina).

#### 2.2.4. Data Analysis

The molecular group described by Doumith et al. [23] was extracted from whole genome sequencing (WGS) data using the scheme described by Moura et al. [24] (https://bigsdb.pasteur.fr/listeria/listeria.html (accessed on 8 September 2021)).

The seven-gene multilocus sequence typing (MLST) scheme, evolutionary lineages, ST, and CC were deducted using the BIGSdb-Lm database (http://bigsdb.pasteur.fr/listeria (accessed on 8 September 2021)). A minimum spanning tree was constructed based on MLST data to show the phylogenetic relationship between the 32 *L. monocytogenes* isolates. A core genome MLST (cgMLST) tree was built using ape and cluster R package. CgMSLT allele matrix for analysis was performed using chewBBACA (https://github.com/B-UMMI/chewBBACA (accessed on 8 September 2021)) using Pasteur Institut cgMLST schema. The software Dendroscope 3 and iTOL 5.5 were used for visualizing the phylogenetic tree.

The whole-genome alignment and clustering were performed using parsnp 1.2-01 software (https://www.ncbi.nlm.nih.gov/pmc/articles/PMC4262987/ (accessed on 1 June 2022). We used *L. monocytogenes* (GeneBank GCA_000196035.1) as a reference genome. For dendrogram visualization, the ggtree R package was applied [25].

Analyzing the position of premature stop codons in obtained sequences facilitated the estimation of the loss of function genes among the analyzed isolates. The total coding sequence (CDS) lengths were shown, starting from transcription initiation to the stop codon (Figure S1). The relative CDS lengths were also calculated as percentage values in the range of 0% to 100% for each sequence. Relative CDS percentages that were set at 0 indicated no detection of the gene at the level of sequencing data, whereas percentages of 100 were considered the full-length CDS based on the UniProt database. Hierarchical clustering and heatmap visualization were performed with the use of the complexheatmap package [26] running in the R environment in version 4.1.

## 3. Results

### 3.1. Analysis of Genetic Diversity of L. monocytogenes Isolates

The *L. monocytogenes* isolates (*n* = 32) were classified to four molecular groups (including serogroups): 15 isolates belonged to group IIa (1/2a-3a) (46.9%), 10 isolates belonged to group IVb (4ab-4b-4d-4e) (31.2%), 4 isolates belonged to group IIc (1/2c-3c) (12.5%), and 3 isolates belonged to group IIb (1/2b-3b-7) (9.4%) (Table 1).

**Figure 1:**
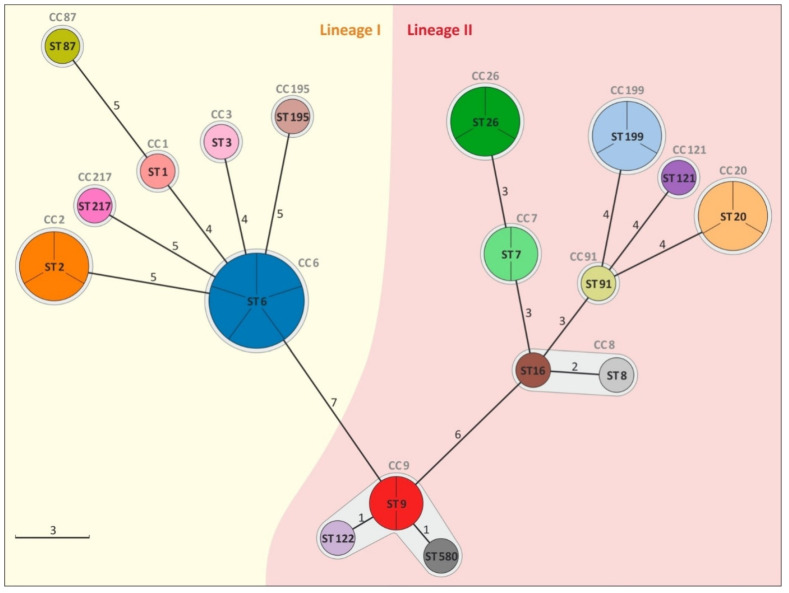
Minimum spanning tree (MST) illustrating the phylogenetic relationship based on sequence types (STs) allelic profiles of *L. monocytogenes* isolates. Each circle represents one ST. The size of the circle is proportional to the number of isolates. The fragment of the circle corresponds to single isolates. Links between the circles are represented according to the number of allelic mismatch between STs.

The isolates were classified into lineage I or lineage II, respectively, containing 40.6% and 59.4% of isolates. A total of 18 different MLST sequence types were identified in the 32 isolates derived from the cheeses: ST 6 (*n* = 5, 15.6% of isolates), ST 2 and ST 20 (each *n* = 3, 9.4%), ST 26 and ST 199 (each *n* = 3, 9.4%), ST 7 and ST 9 (each *n* = 2, 6.3%), and ST 1, ST 3, ST 8, ST 16, ST 87, ST 91, ST 121, ST 122, ST 195, ST 217, and ST 580 (each *n* = 1, 3.1%). Sequence type 7, ST 8, ST 16, ST 20, ST 26, ST 91, ST 121, and ST 199 belonged to serogroup IIa and serovar 1/2a-3a; ST 3, ST 87, and ST 195 belonged to serogroup IIb and serovar 1/2b-3b-7; ST 9, ST 122, and ST 580 belonged to serogroup IIc and serovar 1/2c-3c; and ST 1, ST 2, and ST 6 belonged to serogroup IVb and serovar 4ab-4b-4d-4e. The *L. monocytogenes* isolates were grouped into 15 clonal complexes: CC 6 (*n* = 5), CC 9 (*n* = 4), CC 2, CC 20, CC 26, and CC 199 (each *n* = 3), CC 7 and CC 8 (each *n* = 2), CC 1, CC 3, CC 14, CC 87, CC 121, CC 195, and CC 217 (each *n* = 1) (Table 1). The minimum spanning tree shows that the number of allelic differences between neighboring STs ranged from 1 to 7 (Figure 1).

**Figure 2:**
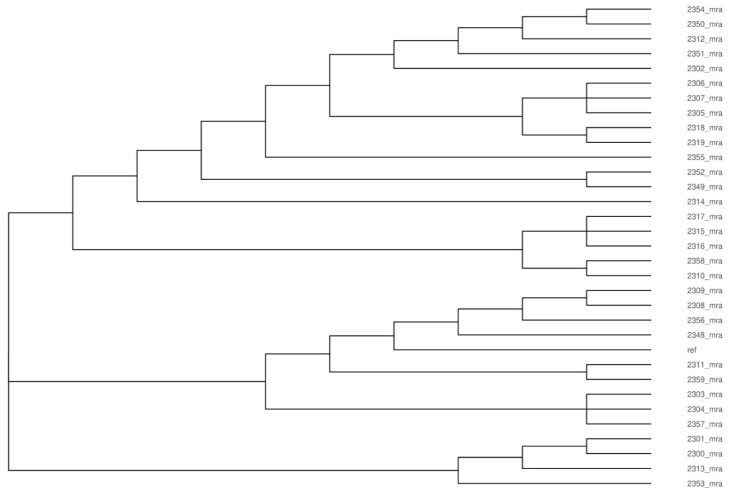
Parsnp dendrogram of the whole-genome alignment showing the similarity of *L. monocytogenes* isolates. All the *Listeria* genomic sequences were aligned to the reference genome, which is labeled as ‘ref’ on the plot.

The parsnp dendrogram of the whole-genome alignment shows the similarity of sequenced *Listeria* isolates on the genomic scale. The total coverage among all the sequences reached 82.2%. The dendrogram shows that the analyzed isolates formed three main clusters that covered 19, 9, and 4 isolates. Isolates belonging to cluster 2 showed the greatest similarity to the reference strain of *L. monocytogenes* (Figure 2).

### 3.2. Analysis of Potential Virulence of L. monocytogenes Isolates

**Figure 3:**
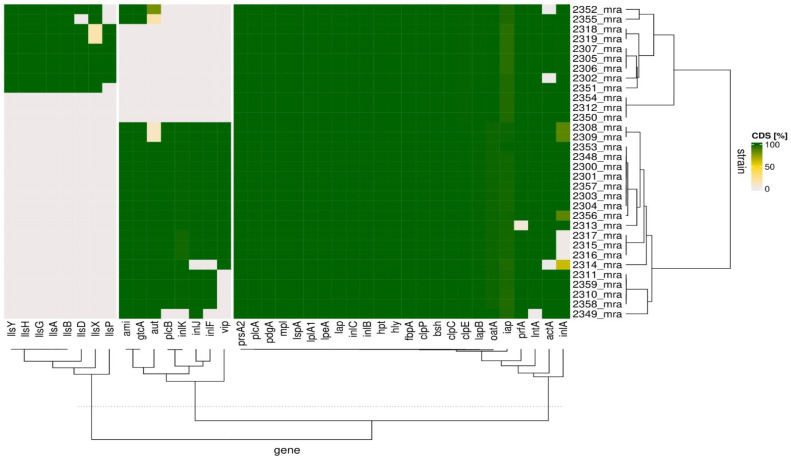
Clustering analysis of virulence genes in *L. monocytogenes* isolates. The heatmap shows the presence of the selected virulence gene set among the analyzed isolates. Normalized coding sequence (CDS) lengths are shown in colors.

Based on the BIGSdb-Lm database, we selected 40 genes reported previously to be important for the virulence of *Listeria* to elucidate the potential virulence of the isolates (Figure 3). The heatmap shows that the analyzed isolates clustered hierarchically into two groups according to the coding sequence (CDS) length of the virulence genes. One of these groups (cluster 1) contained twelve isolates, and the other group (cluster 2) contained twenty isolates. With only a few exceptions, the *Listeria* isolates showed the presence of rather uniform and full CDS-length *prsA2*, *prfA*, *plcA*, *pdgA*, *oatA*, *mpl*, *lspA*, *lplA1*, *lpeA*, *lntA*, *lapB*, *lap*, *inlC*, *inlB*, *inlA*, *iap*, *hpt*, *hly*, *fbpA*, *clpP*, *clpE*, *clpC*, *bsh*, and *actA* genes. These exceptions were that some isolates’ *inlA* copies were judged to be non-functional because of premature stop codon insertion; this applied to more than a fifth (7 out of 32) of the isolates (2308_mra, 2309_mra, 2314_mra, 2315_mra, 1216_mra, 2317_mra, and 2356_mra); *actA* was detected as truncated in three isolates (2302_mra, 2314_mra, and 2352_mra); and *prfA* and *lntA* were truncated in the 2313_mra and 2349_mra isolates, respectively. The bacterial isolates that had been grouped as cluster 1 almost ubiquitously lacked functional sequences in their *aut*, *inlF*, *inlJ*, *inlK*, *plcB*, and *vip* genes. In this cluster, only *ami* and *gtcA* were found with full CDS in two isolates, which were 2352_mra and 2355_mra. Among the isolates that had been grouped in cluster 2, a truncated *aut* gene was present in the 2308_mra and 2309_mra isolates. A set of truncated *inlF*, *inlK*, *plcB*, and *vip* genes was specific only to the 2349_mra isolate in cluster 2. The isolate labeled as 2314_mra in this cluster was negative for *inlF* and *inlJ*. Additionally, five isolates of the same cluster were also negative for *vip* CDS, and these isolates were 2310_mra, 2311_mra, 2349_mra, 2358_mra, and 2359_mra; cluster 2 isolates were also negative for genes that code listeriolysins which had been reported as specific for *Listeria innocua*, namely *llsA*, *llsB*, *llsD*, *llsG*, *llsH*, *llsP*, *llsX*, and *llsY*, whereas the complete CDS of most of the studied listeriolysin genes were present in cluster 1 isolates. In cluster 1, only three isolates (2312_mra, 2350_mra, and 2354_mra) were negative for all of these listeriolysin-coding genes. The absence of *llsP* characterized three other isolates (2351_mra, 2352_mra, and 2355_mra), and *llsX* was not present in 2318_mra or 2319_mra. Additionally, the 2355_mra isolate was negative for the *llsD* gene.

## 4. Discussion

### 4.1. Genetic Diversity of L. monocytogenes Isolates

A total of 32 *L. monocytogenes* isolates originating from artisanal cheeses were typed and characterized for the presence of selected virulence genes. The majority of isolates were classified into group IIa (1/2a-3a), the next group by size was IVb (4ab-4b-4d-4e), then came IIc (1/2c-3c), and the smallest was IIb (1/2b-3b-7). These results are consistent with an analysis of 1698 food-derived isolates, which showed the dominance of the IIa and high representation of the IVb molecular groups among various food matrices, including milk and milk products [6]. In turn, Espinosa–Mata et al. (2022) and Barría et al. (2020) reported that group IVb dominated among *L. monocytogenes* isolates originating from artisanal cheeses [13,15]. Therefore, it seems reasonable to conclude that the potential for *L. monocytogenes* to contaminate food is not limited to one molecular group [6]. Furthermore, analysis of *L. monocytogenes* from artisanal cheeses revealed the presence of isolates from different molecular groups (IIa and IVb) in the same batch of cheese, as well as the presence of isolates from different molecular groups (IIb and IIc) in different products from the same production site, which could suggest coexisting sources of pathogen contamination in these cases (data not shown). Additionally, it should be noted that *L. monocytogenes* belonging to groups IIa, IVb, and IIb were responsible for human infections [7], which, to relate this to the present research, indicated a potential risk associated with the consumption of artisanal cheeses.

*Listeria monocytogenes* strains form a structured population and are differentiated into distinct evolutionary lineages, i.e., I, II, III, and IV, which represent different ecological, genetic, and phenotypic characteristics [27]. Most human listeriosis outbreaks are associated with lineage I isolates, while lineage II strains are common in foods. Lineage II strains are widespread in natural and farm environments and are also commonly isolated from animal listeriosis cases and sporadically isolated from human clinical cases. In contrast, lineage III and IV strains are rare and are predominantly isolated from animal sources [27]. Isolates from artisanal cheeses belonged to lineages I and II in 40.6% and 59.4% proportions, respectively, indicating a significant number of them from lineage I in the pool of isolates. Therefore, a perception of overrepresentation of lineage I strains in sporadic listeriosis cases in proportion to their prevalence in food appears inconsistent with the evidence from these products [27].

Artisanal cheese isolates were revealed to harbor a wide range of sequence types (18 ST having been detected), and those types were shown to have dissimilar prevalences (ST 6 being dominant), geographical distributions (ST 2 occurring in two administrative regions), and frequent co-occurrence in products from the same cheese dairy (ST 6, ST 26, and ST 122 being detected in dairy I; ST 7 and ST 16 in dairy F; ST 195 and ST 580 in dairy C; ST 1 and ST 20 in dairy G_1_; and ST 3 and ST 9 in dairy G_2_) (Table 1). The existence of an MLST database enables data comparison at the international level [28]. The comparison showed that all STs found in artisanal cheeses were known and had previously been reported in certain countries (http://bigsdb.pasteur.fr/listeria (accessed on 8 September 2021)). According to the available literature, 16 ST were found in various foodstuffs, and they were ST1, ST2, ST3, ST6, ST7, ST8, ST9, ST16, ST20, ST26, ST87, ST91, ST121, ST122, ST217, and ST 580 [8,29,30,31,32]. It is significant that some of these STs have been associated with listeriosis (ST 1, ST 2, ST 3, ST 6, ST 7, ST 8, ST 9, ST 20, ST 26, ST 87, and ST 121) [8,33,34,35] and epidemics (ST 6 and ST 87) [36,37]. It is also on record that ST 195 was isolated from clinical cases [6]. Artisanal cheese isolates were also classified into 15 CC (Table 1; Figure 1). In the presented studies, the dominant CC was CC 6, which, according to the results of studies by other authors, was more often attributed to clinical cases than detected in food [38]. This CC was regarded as rare until 2000, while since that year, its relative frequency has increased [7]. In the same time interval, a similar trend was observed for CC 8, CC 9, and CC 121, while the relative frequency of CC 1, CC 2, CC 3, CC 7, CC 14, CC 20, and CC 199 was lower [7]. The frequency distribution of *L. monocytogenes* CC in food and clinical sources is highly uneven, and three categories of highly prevalent complexes can be distinguished: infection-associated (CC 1, CC 2, CC 4, and CC 6), food-associated (CC 9 and CC 121), and intermediate (others) [38]. The results of the presented studies showed that there were CCs from these three categories in the artisanal cheeses. It should be noted that, with the exception of CC 199, the isolates identified in these studies (totaling 14: CC 1, CC 2, CC 3, CC 6, CC 7, CC 8, CC 9, CC 14, CC 20, CC 26, CC 87, CC 121, CC 195, and CC 217) have had clinical cases attributed to them in many countries around the world [8,38,39]. In addition, the artisanal cheeses studied contained complexes from among the most common CCs in Europe (CC 2, CC 6, CC 7, and CC 9) [8].

Multiple alignments of analyzed genomes indicated the close evolutionary relationship of isolated *L. monocytogenes*. Neither the administrative division, the cheese dairy, nor the year of isolation had relevance to the extent to which *Listeria monocytogenes* isolates were related (Figure 2).

### 4.2. Virulence Genes of L. monocytogenes Isolates

According to the microbiological criterion for *L. monocytogenes* in food [40], all strains of this pathogen are considered equally virulent. However, differences in pathogen virulence have been described in the subject literature [38,41]. The isolates from artisanal cheeses were compared for selected 40 virulence genes (Figure 3 and Figure S1) that affect the intracellular life cycle of *L. monocytogenes*, i.e., host cell adhesion and invasion, intracellular multiplication and motility, and intercellular spread [42].

The *L. monocytogenes* genome includes a large family of genes expressing proteins known as internalins (Inl) [43], which are important in the early stages of infection. Literature data showed that beyond the extensively-studied invasins *inlA* and *inlB* (considered species-specific), other internalins such as *inlF*, *inlJ*, *inlC*, and *inlK* also fulfill important functions in the infectious process. The internalin genes are involved in adherence (*inlF* and *inlJ*), cell-to-cell spread (*inlC*), and autophagy evasion (*inlK*) [43,44,45,46,47]. The *inlA*, *inlB,* and *inlC* genes were found in all isolates of lineages I and II (32/32), but some isolates of lineage II had premature stop codons (PMSC) in the *inlA* gene (7/19), which suggests a reduced ability to invade human intestinal epithelial cells in vitro [48]. In contrast, the *inlF* and *inlK* genes were found only in isolates from lineage II (19/19 and 18/19, respectively), whereas the *inlJ* gene was present in isolates from lineage II (18/19) and only 1/13 from lineage I. 

LIPI-1 (*Listeria* pathogenicity island 1) and LIPI-3 are considered to be responsible for the increased virulence in some strains of the pathogen [41]. It is pertinent that all invasive strains but one possessed LIPI-1 [41]. Intracellular pathogenesis heavily relies on factors transcribed by *prfA*, *hly*, *plcA*, *mpl*, *actA*, and *plcB* genes located in the LIPI-1 (*Listeria* pathogenicity island 1) [49], which are all absent from *L. innocua*, the closest non-pathogenic *Listeria* species to *L. monocytogenes* [50]. The *prfA* gene is the major regulator of the virulence gene expression [49]; the *hly* gene encodes listeriolysin O (LLO), which plays a central role in the cell-to-cell spread process of *L. monocytogenes* [51] as well as being involved in several stages of the intracellular lifecycle of the pathogen [52]; the *plcA* and *plcB* genes encode phosphatidylinositol-specific phospholipase C and broad-range phospholipase C, respectively [53], which are necessary at the stage of phagosome lysis and pathogen release into the cytoplasm of host cells; the *mpl* gene encoding metalloprotease is required for *actA* processing and protrusion resolution, besides being involved in *plcB* processing and vacuole escape [54]; and the *actA* gene is an essential virulence factor of *L. monocytogenes* and its extracellular action enhances virulence in contributing to aggregation and biofilm formation to mediate colonization of the gut lumen, promoting and enhancing bacterial host cell entry, facilitating evasion of autophagy and vacuolar exit, as well as activating nuclear factor of kappa light polypeptide gene enhancer in B-cells (NF-κB) [55]. The results of the presented studies show that the *prfA*, *plcA*, *plcB*, *mpl*, and *hly* genes were present in 32/32 and the *actA* gene in 29/32 of isolates. Premature stop codons in the *plcB* and *prfA* genes occurred in all lineage I isolates (13/13) and 1 lineage II isolate (1/19). 

*Listeria monocytogenes* strains may carry the *llsA*, *llsB*, *llsD*, *llsG*, *llsH*, *llsP*, *llsX,* and *llsY* genes, together designated LIPI-3 [5]. Pathogenicity studies in murine models indicated that LIPI-3 was responsible for the increased virulence of some strains of the pathogen [41]. LIPI-3 encodes an additional hemolysin, listeriolysin S (LLS), which is a hemolytic/cytolytic factor impacting potential virulence [56]. LIPI-3 genes were only found in isolates from lineage I (9/13). These genes were found in a set of 8 in 6/13 isolates, a set of 7 in 2/13 (with the *llsP* gene absent), and a set of 6 in 1/13 (with the *llsP* and *llsD* genes absent), which implied these isolates higher potential virulence compared to the other isolates of both lineages at the stage of pathogen release from the phagosome. Furthermore, Vilchis–Rangel et al. [41] pointed to a strong association between *llsX* and the invasiveness of *L. monocytogenes*. The *llsX* gene was found in all nine isolates of this line; however, two isolates had premature stop codons. 

The *ami* gene mediates the adhesion of *L. monocytogenes* to eukaryotic cells via its cell wall–anchoring domain, and its inactivation leads to a severe loss of adhesion [57]. The *gtcA* protein is necessary for lipoteichoic acid glycosylation in *L. monocytogenes* [58]. A mutation in the *gtcA* gene reduced the ability of strains carrying it to invade intestinal epithelial cells under experimental conditions, which may be associated with reduced virulence of those strains [59]. All isolates from lineage II (19/19) and three from lineage I (3/13) contained the *ami* and *gtcA* genes. 

The *aut* gene encodes the auto surface protein with autolytic activity, which is required for the entry of *L. monocytogenes* into eukaryotic cells and contributes to virulence in vivo [60]. Studies in mice and guinea pigs showed significantly less virulence of *L. monocytogenes* with an inactivated *aut* gene [60]. Among the researched *L. monocytogenes*, all isolates of lineage II (19/19) and only three isolates of lineage I (3/13) had the *aut* gene, while two isolates of lineage I and lineage II (2/13 and 2/19) were detected to have premature stop codons in this gene. 

The *vip* gene is a virulence factor only present in pathogenic *Listeria* species, which is required for entry into some mammalian cells and for virulence (for efficient entry into Caco-2 human intestinal epithelial cells and L2071 mouse fibroblast cells, but not into GPC16 guinea pig colon adenocarcinoma or Vero African green monkey kidney cells) [61]. This virulence factor gene is present in all *L. monocytogenes* lineage I and II serovars that include serovars implicated in human disease (1/2a, 1/2c, 1/2b, and 4b) and absent from non-pathogenic species of *Listeria* [44]. Among artisanal cheese isolates, the *vip* gene was found in only 15 isolates from lineage II (15/19). 

The *prsA2* gene plays a unique and important role in *L. monocytogenes* pathogenesis by promoting the activity and stability of at least two critical secreted virulence factors: LLO and a broad-specificity phospholipase. Loss of *prsA2* activity severely attenuated virulence in mice and impaired bacterial cell-to-cell spread in host cells [62]. All artisanal cheese-derived isolates from both lineages had the *prsA2* gene (32/32). 

The *lntA* gene encodes a bacterial nucleomodulin which acts directly in the nucleus to manipulate a chromatin regulatory protein. The secreted virulence factor allows a pathogen to control host chromatin composition, transcription, and gene expression [63]. The *lntA* gene was found in isolates of lineage II (19/19) and lineage I (12/13). 

The *iap* gene of *L. monocytogenes* encodes the extracellular protein p60 (a murein hydrolase), which is necessary for septum separation and for the successful invasion of host cells [42]. The *iap* gene was found in lineage I and lineage II isolates (32/32). 

The *bsh* gene determines the production of bile salt hydrolase, which is a protein conferring on *L. monocytogenes* resistance to the action of bile salts, and its production increases the chance of survival of the pathogen in the gastrointestinal tract [64]. The *bsh* gene was present in all isolates from artisanal cheeses from lineages I and II (32/32). 

The genes encoding stress proteins, i.e., *clpP*, *clpC,* or *clpE,* are crucial to *L. monocytogenes* at the stage of intracellular growth. The *clpP* gene encodes a protein that is involved in the rapid adaptive response of intracellular pathogens during the infectious process [65]. The *clpC* gene encodes a virulence factor promoting the intracellular survival of *L. monocytogenes* by facilitating its early bacterial escape from the phagosomal compartment of a macrophage [66]. It is also required for adhesion and invasion, and it governs InlA, InlB, and ActA expression [67]. The ClpE protein acts synergistically with ClpC in cell septation [68]. Active *clpP*, *clpC,* and *clpE* genes were found in all isolates from lineages I and II (32/32). 

The *pdgA* and *oatA* genes encode peptidoglycan N-deacetylase (PdgA) and peptidoglycan O-acetyltransferase (OatA), respectively, by which *L. monocytogenes* acquires resistance to antibacterial compounds of which the action is directed at the cell wall of the pathogen, including lysozyme. The resistance is gained by modifying the structure of peptidoglycans, and these peptidoglycan modifications are essential for the survival of the pathogen in macrophages. In addition, PdgA and OatA enable *L. monocytogenes* to suppress the cytokine response of the infected organism. The *pdgA* and *oatA* genes are important factors used by *L. monocytogenes* for the effective colonization of host cells [69]. The *pdgA* and *oatA* genes were found in all isolates from lineages I and II (32/32). 

The *hpt* gene encodes the transporter protein (Hpt) that *L. monocytogenes* exploits to be able to use glucose-1-phosphate from the host cell as a source of carbon and energy at the stage of intracellular multiplication. In in vivo experiments in mice, the absence of the *hpt* gene in *L. monocytogenes* impaired the bacterium’s proliferation within the host cell and decreased its virulence [3,42]. The *hpt* gene was found in all isolates from lineages I and II (32/32). 

The *lap* gene encodes the Lap adhesion protein, which, when bound to the host cell receptor, enhances the adhesion of *L. monocytogenes* to intestinal epithelial cells [42]. All isolates of *L. monocytogenes* from artisanal cheeses (32/32) carried the *lap* gene. 

The *lapB* gene encodes the LapB adhesion protein characteristic only of pathogenic species of *Listeria*. This protein is essential for adhesion to and penetration into host intestinal epithelial cells by *L. monocytogenes* [42]. All isolates of *L. monocytogenes* from artisanal cheeses (32/32) also had the *lapB* gene. 

The *fbpA* gene encodes the production of fibronectin-binding protein (FbpA), which increases the adhesion of *L. monocytogenes* to intestinal epithelial cells and acts as a chaperone protein to stabilize and/or ensure proper secretion of LLO and InlB [42]. All isolates of *L. monocytogenes* from artisanal cheeses (32/32) were found to have the *fbpA* gene. 

The *lspA* gene encodes a signal peptidase in class II (SPase II), which is a co-actor in the maturation of lipoproteins in *L. monocytogenes* infection. The maturation of lipoproteins (from a precursor form to mature protein) is crucial for the surface protein anchoring and effective phagosome escape and intracellular survival of *L. monocytogenes* [70]. All isolates of *L. monocytogenes* from artisanal cheeses (32/32) carried the *lspA* gene. 

The *lpeA* gene is responsible for the synthesis of the lipoprotein promoting entry A (LpeA) necessary for *L. monocytogenes* to penetrate eukaryotic cells and acts to facilitate intracellular survival of the pathogen in infected macrophages. Mutated *L. monocytogenes* cells with *lpeA* deletion failed to infect human Caco-2 cells or mouse hepatocytes (tib73) in cell cultures [42,71]. The *lpeA* gene was found in all isolates (32/32). 

The *lplA1* gene encodes lipoate protein ligase (LplA1) necessary for intracellular replication of *L. monocytogenes*. LplA1 allows the pathogen to use lipoic peptides derived from the cytosol of host cells as a source of lipoate, which promotes the adaptation of *L. monocytogenes* to living in infected mammalian cells [72]. The *lplA1* gene was present in all isolates of *L. monocytogenes* from artisanal cheeses (32/32). 

In general, the comparison of 32 artisanal cheese isolates with respect to 40 selected virulence genes showed a greater diversity of isolates between lineages than within the same lineage. In contrast to lineage I isolates, those of lineage II did not have LIPI-3 genes, which confirms that lineage II strains may be more adapted to an environmental lifestyle than lineage I strains [27]. An additional difference between lineage I and lineage II involved the *vip*, *inlF,* and *inlK* genes, which were only present in lineage II isolates. This may be related to the diverging evolutionary pathways followed by virulence genes, which are affected by a strain’s origin and serotype [73]. A common feature of isolates from both lineages was the presence of most LIPI-1 genes (*prfA*, *plcA*, *mpl*, *hly*, *plcB,* and *actA*) and the presence of the *inlA*, *inlC*, *inlB*, *prsA2*, *iap*, *bsh*, *clpP*, *clpE*, *clpC*, *pdgA*, *oatA*, *hpt*, *lap*, *lapB*, *fbpA*, *lspA*, *lpeA*, and *lplA1* genes. Bechtel and Gobbons [74] reported that some *L. monocytogenes* populations may associate with particular foods, including cheese, and that gene content may contribute to this pattern, which may, to some extent, explain the observed similarities and differences in genes among artisanal cheese isolates.

## 5. Conclusions

The studies provided information on the genetic diversity and selected virulence genes of the isolates of *L. monocytogenes* derived from specific dairy products, i.e., artisanal cheeses. The populations of *L. monocytogenes* isolated from the cheeses were differentiated in terms of molecular groups (including serogroups), evolutionary lineages, sequence types, clonal complexes, and potential virulence. The small number of isolates obtained from artisanal cheeses limits the representativeness of the results; however, the presence in this foodstuff of isolates from lineages I and II in comparable numbers suggests that attributing dominance to lineage II in this type of food is unjustified. The complete absence of lineage III and IV isolates is consistent with the observation that these two lineages are rarely isolated from food-related environments. The sequence types and clonal complexes to which the isolates from the cheeses belonged were distributed locally and globally. The study also confirmed that premature stop codon mutations in *inlA* and the absence of LIPI-3 are characteristic of lineage II isolates. In turn, the presence of premature stop codon mutations in the *plcB* gene and the absence of the *vip* gene in all isolates from lineage I appear to be characteristic of isolates of this line. The administrative division, cheese dairy, or year of isolation had no relevance to the extent to which *L. monocytogenes* isolates were related.

Artisanal cheeses can carry virulent strains of *L. monocytogenes*. Products originating in the same batch (produced in the same cheese dairy, under the same conditions, and at the same time) can simultaneously harbor isolates from different molecular groups, including serovars, different evolutionary lineages, different sequence types, different clonal complexes, and of different virulences, and that dairy can be a place where strains classified into different ST and CC are in circulation.

Relating the presented studies to food safety, the effective treatment of listeriosis, and potential new scientific research, they indicate an area for further research on the sensitivity to disinfectants and antibiotics of isolates from artisanal cheeses.

## Figures and Tables

**Table 1:** Distribution (administrative divisions and cheese dairies), numbers, molecular groups, lineages, allelic profiles (ST), clonal complexes (CC) of the *L. monocytogenes* isolates.

Administrative Divisions	Cheese Dairy	Number of Isolates *n* = 32	Year of Isolation	Molecular Group	Lineage	ST	CC
2	D_1_, D_2_, I	5	2016, 2018	IV b 4ab-4b-4d-4e	I	6	6
2	D_3_, G_1_	3	2014, 2018	II a 1/2a-3a	II	20	20
2, 3	G_3_, J	3	2015, 2017, 2018	IV b 4ab-4b-4d-4e	I	2	2
2	I	3	2015	II a 1/2a-3a	II	26	26
2	H	3	2018	II a 1/2a-3a	II	199	199
2	G_2_	2	2016	II c 1/2c-3c	II	9	9
2	F	2	2017	II a 1/2a-3a	II	7	7
2	G_1_	1	2014	IV b 4ab-4b-4d-4e	I	1	1
2	F	1	2018	II a 1/2a-3a	II	16	8
4	K	1	2018	II a 1/2a-3a	II	121	121
2	I	1	2018	II c 1/2c-3c	II	122	9
2	D_4_	1	2014	IIb 1/2b-3b-7	I	87	87
1	A	1	2016	IV b 4ab-4b-4d-4e	I	217	217
1	C	1	2016	II b 1/2b-3b-7	I	195	195
2	E	1	2018	II a 1/2a-3a	II	91	14
2	G_2_	1	2017	II b 1/2b-3b-7	I	3	3
1	C	1	2017	II c 1/2c-3c	II	580	9
1	B	1	2018	II a 1/2a-3a	II	8	8

1–4 administrative divisions/A–K cheese dairy: 1/A, B, C; 2/D, E, F, G, H, I; 3/J; 4/K. Different capital letters (from A to K) indicate the production sites in different towns, while the same capital letters with different subscripts (D_1_–D_4_, G_1_–G_3_) show different production sites in one town. ST—sequence type; CC—clonal complex.

## Data Availability

The data of the present study are available from the corresponding author upon request.

## Supplementary Materials

The following supporting information can be downloaded at: https://www.mdpi.com/article/10.3390/foods11182805/s1, Figure S1: Diverging graphs of coding sequence length among analyzed Listeria isolates. The absolute length in base pairs (bp) of the gene is shown for all of the sequenced Listeria isolates. The CDS length range for a selected gene is color-coded dark blue (minimum length) to light blue (maximum length).
