# Supplementary material for: Genetic Diversity and Potential Virulence of Listeria monocytogenes Isolates Originating from Polish Artisanal Cheeses
Source: Foods. 2022 Sep 11;11(18):2805. doi: 10.3390/foods11182805 (PMC9497517; doi:10.3390/foods11182805)
Supplement: Supplementary file 1 [file foods-11-02805-s001.zip › foods-1850081-supplementary-Figure S1.pdf]

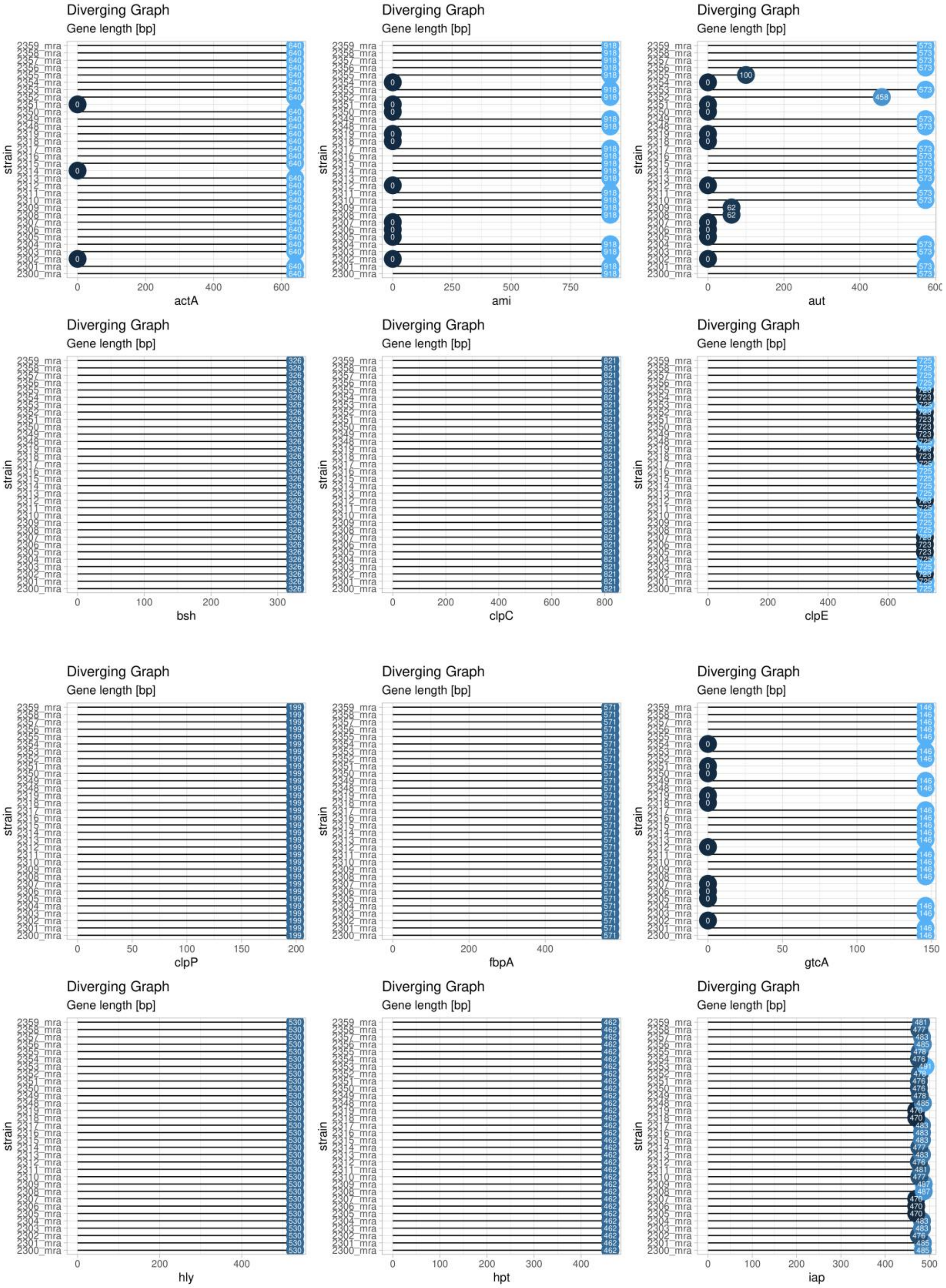
Diverging Graph
Gene length [bp]
strain
actA
Diverging Graph
Gene length [bp]
strain
ami
Diverging Graph
Gene length [bp]
strain
aut
Diverging Graph
Gene length [bp]
strain
bsh
Diverging Graph
Gene length [bp]
strain
clpC
Diverging Graph
Gene length [bp]
strain
clpE
Diverging Graph
Gene length [bp]
strain
clpP
Diverging Graph
Gene length [bp]
strain
fbpA
Diverging Graph
Gene length [bp]
strain
gtcA
Diverging Graph
Gene length [bp]
strain
hly
Diverging Graph
Gene length [bp]
strain
hpt
Diverging Graph
Gene length [bp]
strain
iap

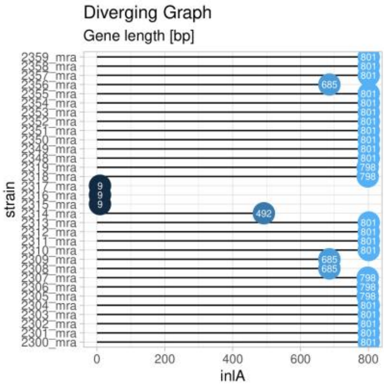
Diverging Graph
Gene length [bp]
strain
0 200 400 600 800
inlA

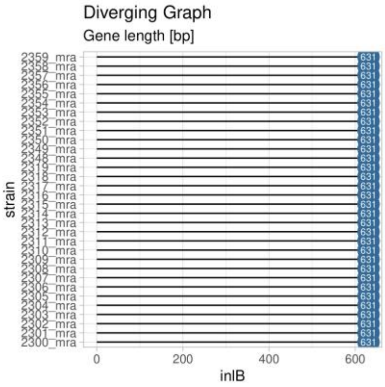
Diverging Graph
Gene length [bp]
strain
0 200 400 600
inlB

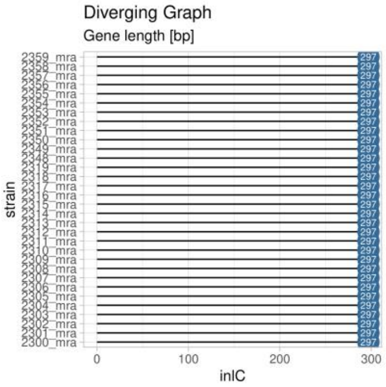
Diverging Graph
Gene length [bp]
strain
0 100 200 300
inlC

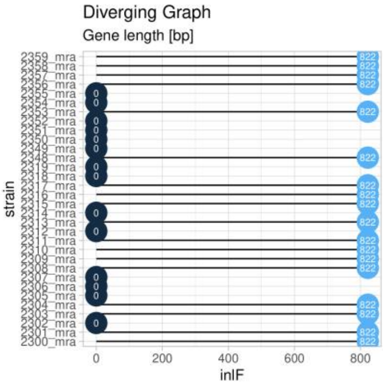
Diverging Graph
Gene length [bp]
strain
0 200 400 600 800
inlF

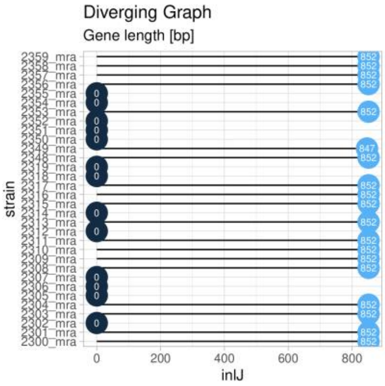
Diverging Graph
Gene length [bp]
strain
0 200 400 600 800
inlJ

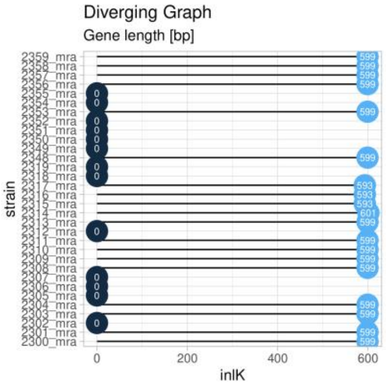
Diverging Graph
Gene length [bp]
strain
0 200 400 600
inlK

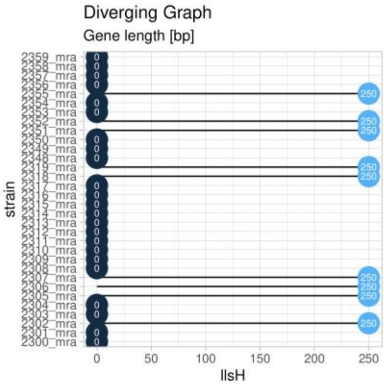
Diverging Graph
Gene length [bp]
strain
0 50 100 150 200 250
llsH

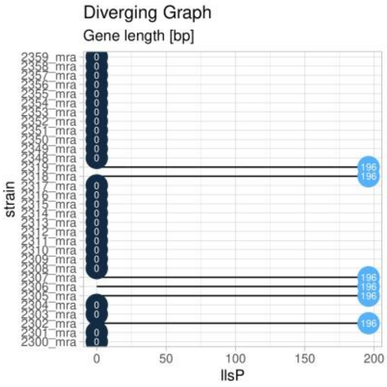
Diverging Graph
Gene length [bp]
strain
0 50 100 150 200
llsP

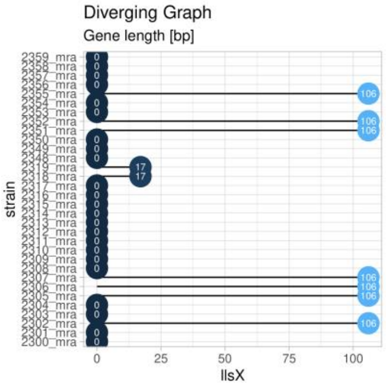
Diverging Graph
Gene length [bp]
strain
0 25 50 75 100
llsX

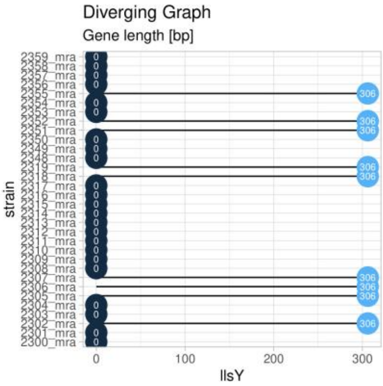
Diverging Graph
Gene length [bp]
strain
0 100 200 300
llsY

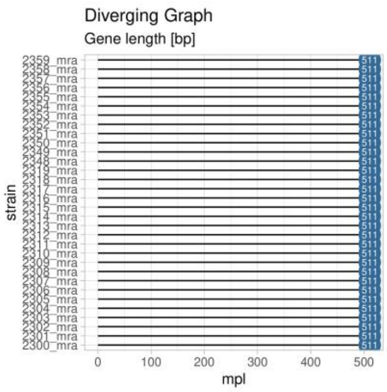
Diverging Graph
Gene length [bp]
strain
511
0 100 200 300 400 500
mpl

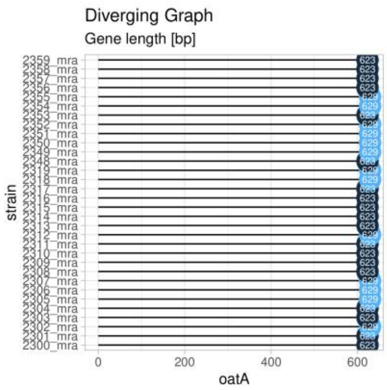
Diverging Graph
Gene length [bp]
strain
623
629
0 200 400 600
oatA

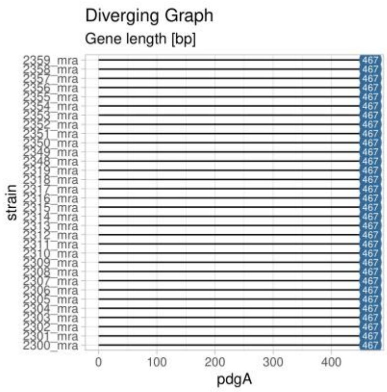
Diverging Graph
Gene length [bp]
strain
467
0 100 200 300 400
pdgA

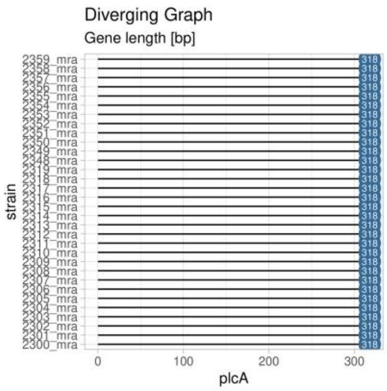
Diverging Graph
Gene length [bp]
strain
318
0 100 200 300
plcA

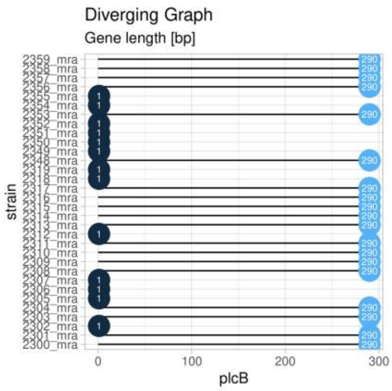
Diverging Graph
Gene length [bp]
strain
290
1
0 100 200 300
plcB

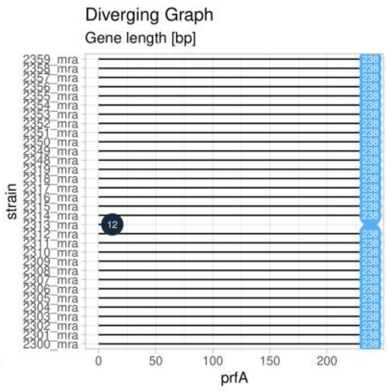
Diverging Graph
Gene length [bp]
strain
238
12
0 50 100 150 200
prfA

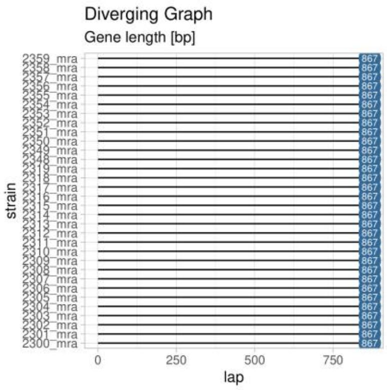
Diverging Graph
Gene length [bp]
strain
867
0 250 500 750
lap

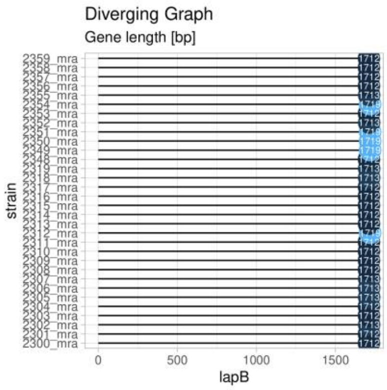
Diverging Graph
Gene length [bp]
strain
1712
1719
0 500 1000 1500
lapB

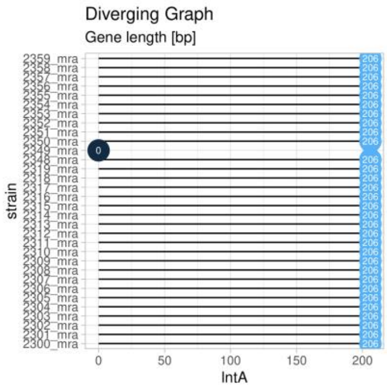
Diverging Graph
Gene length [bp]
strain
206
0
0 50 100 150 200
lntA

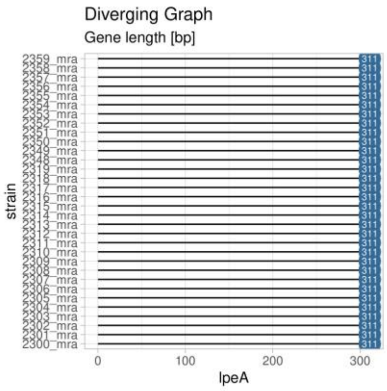
Diverging Graph
Gene length [bp]
strain
311
0 100 200 300
lpeA

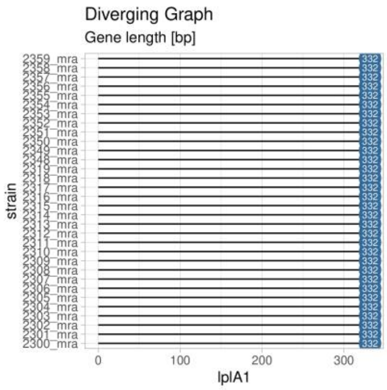
Diverging Graph
Gene length [bp]
strain
332
0 100 200 300
lplA1

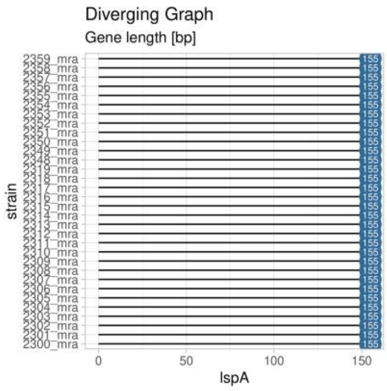
Diverging Graph
Gene length [bp]
strain
155
0 50 100 150
lspA

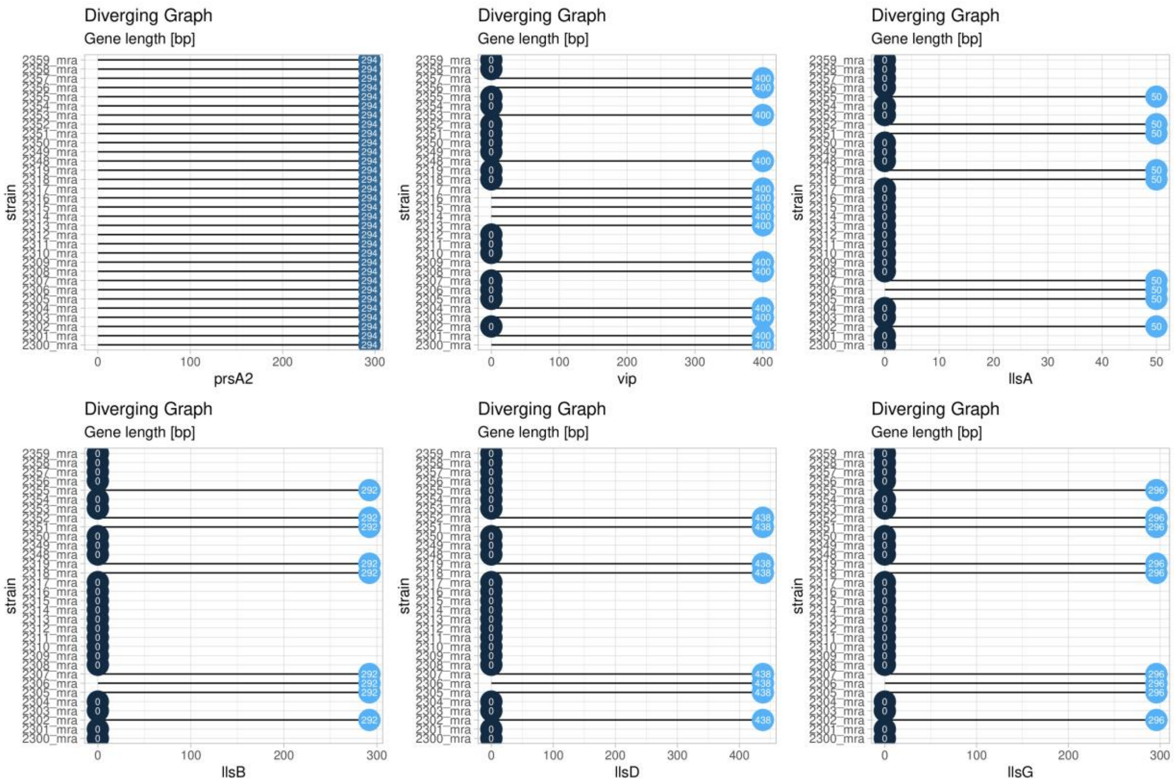

Figure S1 Diverging graphs of coding sequence length among analyzed *Listeria* isolates. The absolute length in base pairs (bp) of the gene is shown for all of the sequenced *Listeria* isolates. The CDS length range for a selected gene is color-coded dark blue (minimum length) to light blue (maximum length).
